# Neuroinflammation, cerebrovascular dysfunction and diurnal cortisol biomarkers in a memory clinic cohort: Findings from the Co-STAR study

**DOI:** 10.1038/s41398-024-03072-x

**Published:** 2024-09-09

**Authors:** Makrina Daniilidou, Jasper Holleman, Göran Hagman, Ingemar Kåreholt, Malin Aspö, Ann Brinkmalm, Henrik Zetterberg, Kaj Blennow, Alina Solomon, Miia Kivipelto, Shireen Sindi, Anna Matton

**Affiliations:** 1 Division of Clinical Geriatrics, Center for Alzheimer Research, Department of Neurobiology, Care Sciences and Society, Karolinska Institutet, Stockholm, Sweden; 2 Division of Neurogeriatrics, Center for Alzheimer Research, Department of Neurobiology, Care Sciences and Society, Karolinska Institutet, Stockholm, Sweden; 3 Theme Inflammation and Aging, Karolinska University Hospital, Stockholm, Sweden; 4 Institute of Gerontology, School of Health and Welfare, Jönköping University, Jönköping, Sweden; 5 Department of Psychiatry and Neurochemistry, Institute of Neuroscience and Physiology, the Sahlgrenska Academy at the University of Gothenburg, Mölndal, Sweden; 6 Clinical Neurochemistry Laboratory, Sahlgrenska University Hospital, Mölndal, Sweden; 7 Department of Neurodegenerative Disease, UCL Institute of Neurology, Queen Square, London, UK; 8 UK Dementia Research Institute at UCL, London, UK; 9 Hong Kong Center for Neurodegenerative Diseases, Clear Water Bay, Hong Kong, China; 10 Wisconsin Alzheimer’s Disease Research Center, University of Wisconsin School of Medicine and Public Health, University of Wisconsin-Madison, Madison, WI USA; 11 Ageing Epidemiology Research Unit (AGE), School of Public Health, Faculty of Medicine, Imperial College London, London, UK; 12 Institute of Clinical Medicine, Neurology, University of Eastern Finland, Kuopio, Finland; 13 Institute of Public Health and Clinical Nutrition, University of Eastern Finland, Kuopio, Finland

**Keywords:** Psychiatric disorders, Diagnostic markers

## Abstract

Cortisol dysregulation, neuroinflammation, and cerebrovascular dysfunction are biological processes that have been separately shown to be affected in Alzheimer’s disease (AD). Here, we aimed to identify biomarker signatures reflecting these pathways in 108 memory clinic patients with subjective cognitive decline (SCD, N = 40), mild cognitive impairment (MCI, N = 39), and AD (N = 29). Participants were from the well-characterized Cortisol and Stress in Alzheimer’s Disease (Co-STAR) cohort, recruited at Karolinska University Hospital. Salivary diurnal cortisol measures and 41 CSF proteins were analyzed. Principal component analysis was applied to identify combined biosignatures related to AD pathology, synaptic loss, and neuropsychological assessments, in linear regressions adjusted for confounders, such as age, sex, education and diagnosis. We found increased CSF levels of C-reactive protein (CRP), interferon γ-inducible protein (IP-10), thymus and activation-regulated chemokine (TARC), intercellular adhesion molecule-1 (ICAM-1), and vascular cell adhesion molecule-1 (VCAM-1) in MCI patients. Further, markers of cortisol dysregulation (flattened salivary cortisol awakening response and flattened cortisol slope) correlated with increased levels of placental growth factor (PlGF), IP-10, and chitinase 3-like 1 (YKL-40) in the total cohort. A biosignature composed of cortisol awakening response, cortisol slope, and CSF IL-6 was downregulated in AD patients. Moreover, biomarker signatures reflecting overlapping pathophysiological processes of neuroinflammation and vascular injury were associated with AD pathology, synaptic loss, and worsened processing speed. Our findings suggest an early dysregulation of immune and cerebrovascular processes during the MCI stage and provide insights into the interrelationship of chronic stress and neuroinflammation in AD.

## Introduction

Alzheimer’s disease (AD) is the most common form of dementia, characterized by the accumulation of extracellular amyloid β plaques and the deposition of intracellular hyperphosphorylated tau lesions in the brain [1, 2]. The ATN (amyloid β, pathologic tau, and neurodegeneration) biomarkers are currently used for a biological definition of AD, according to the 2018 National Institute on Aging and Alzheimer’s Association (NIA-AA) research framework [3]. In addition, CSF markers of synaptic loss such as neurogranin (NG), synaptosomal-associated protein-25 (SNAP-25), and synaptotagmin-1 (SYT-1) have emerged as potential AD biomarkers as they are consistently elevated along the AD continuum [4, 5] and seem to reflect both amyloid β and tau pathology [6–8]. Despite these known alterations that define AD, there are still other pathophysiological pathways that are involved in its development. A better characterization of these mechanisms can provide a deeper understanding of the complex pathology of AD and assist in more effective preventive interventions and disease-modifying treatments [9].

Accumulating evidence suggests that dysregulation of cortisol secretion is implicated in AD [10]. Cortisol, one of the most important mediators of stress in humans, is released by the adrenal cortex to promote adaptation toward a stressor [11]. Its secretion follows a diurnal pattern characterized by a peak (50–60%) 30–45 min after waking (called the cortisol awakening response or CAR), and a subsequent decline over the remainder of the day, reaching lowest levels around midnight [12]. Glucocorticoids can freely pass the blood-brain barrier (BBB) where they exert their brain function through binding to their receptors, located in the hippocampus and other brain regions [13, 14]. In older adults, elevated cortisol levels have been shown to predict hippocampal atrophy and worsened memory [15]. Furthermore, cortisol is increased in AD patients [16, 17], correlates with disease severity [18], and predicts faster cognitive decline and increased AD risk [17]. Our group showed recently that specific diurnal cortisol patterns are associated with cognitive function and AD biomarkers in memory clinic patients of the Cortisol and Stress in Alzheimer’s Disease (Co-STAR) study when stratified by amyloid β status [19].

Microglia and astrocyte activation are common features in AD, resulting in the release of several mediators of inflammation in the brain parenchyma [20]. Numerous studies have reported alterations in the levels of pro-inflammatory cytokines and other modulators of inflammation in the CSF of mild cognitive impairment (MCI) and AD patients [20–22]. Interestingly, microglial activation has been observed even at the pre-plaque stages in both human and animal studies [23, 24] and it is suggested to be adaptive at initial stages and detrimental as AD progresses [20]. Stress response and inflammation are two systems that are tightly correlated to one another. While cortisol exerts its functions as an anti-inflammatory agent through well-known mechanisms, it can also promote inflammation [25]. In fact, chronic exposure to cortisol has been shown to activate nucleotide-binding domain, leucine-rich–containing family, pyrin domain–containing-3 (NLRP3), a critical component of the inflammasome [26] and to induce the release of proinflammatory molecules in the periphery [27]. In addition, Sudheimer et al. [28] showed a synergistic effect of elevated waking cortisol with IL-1β (interleukin 1β) and tumor necrosis factor α (TNF-α) on reduced hippocampal volume in healthy older adults.

At the same time, alterations in cerebrovascular function are not only tightly linked to neuroinflammation but also to neurodegeneration and AD progression [29]. Indeed, data support that cerebrovascular damage compromises the clearance of amyloid β [29], while vascular dysfunction factors are often found increased in the CSF throughout the AD continuum and are associated with AD pathology markers [30].

Despite this evidence, less is known about the combined effect of cortisol with neuroinflammation and cerebrovascular dysfunction in the context of AD, and to the best of our knowledge there are no human studies that have previously explored these systems together in AD. The aim of this work was to identify specific biomarker signatures reflecting these pathways in patients with neurocognitive disorders. Furthermore, we aimed to explore biosignature relationships with biomarkers of AD pathology and synaptic loss as well as cognitive performance and other neuropsychological features (depressive symptoms, perceived stress). We addressed these research questions by studying cross-sectionally a broad panel of biomarkers in patients with subjective cognitive decline (SCD), MCI, and AD from the Co-STAR cohort.

## Subjects and Methods

### Study design and participants

This research is based on the Co-STAR cohort study. A detailed description of the study design has been published elsewhere [19]. In brief, participants were recruited from the Karolinska University Hospital memory clinic in Huddinge, Sweden between 2014 and 2017. The inclusion criteria were individuals with a diagnosis of SCD, MCI or dementia, aged 45+ years, who were physically able to participate. Exclusion criteria were conditions affecting the HPA-axis (e.g. Cushing syndrome). Six hundred forty-nine patients were approached at their first memory clinic visit, from which 280 did not fulfill these inclusion criteria, while a further 136 declined to participate. In total, 233 participants agreed to participate, 188 of whom provided data and did not withdraw their consent to be included. Patients under cortisone treatment or with a dementia diagnosis other than AD, were further excluded from this study. Of these, 108 had sufficient data for the assessment of diurnal cortisol patterns, CSF biomarkers, and cognitive function.

### Clinical assessments

The clinical assessment at the memory clinic has been described in detail elsewhere [19]. Briefly, it was performed through a harmonized diagnostic evaluation consisting of a comprehensive medical and neurologic examination, medical and informant-based history, neuropsychological evaluation, blood chemistry, MRI, *APOE* genotyping, and CSF biomarker analysis (Aβ42, phospho-tau (p-tau) 181 and total-tau (t-tau)). A multidisciplinary team evaluated each patient and set a consensus diagnosis based on all test results, including biomarkers. AD diagnosis was based on the *International Classification of Diseases, Tenth Revision (ICD-10)* [31] and the Winblad et al. criteria were used for MCI diagnosis [32]. Patients who did not meet the criteria for MCI or dementia were considered to experience SCD.

### Cognitive assessments

Two cognitive domains were included in this study: memory and processing speed. A detailed description of the assessments has been published elsewhere [19]. Briefly, the memory score was based on the Rey Auditory Verbal Learning Test (delayed recall) [33], the Rey-Osterrieth Complex Figure (immediate recall) [34] the Wechsler Adult Intelligence Scale (WAIS) Digit Symbol Substitution Test (immediate recall) [35] and the Hagman test, developed at the Karolinska University Hospital to assess visual memory (manuscript in preparation). Processing speed was assessed with the WAIS Digit Symbol Substitution Test. Unadjusted z-scores were calculated for all tests, and those related to the same cognitive domain were averaged to create cognition scores.

### Salivary Cortisol

Salivary cortisol was quantified as previously published [19]. Briefly, participants were given home sampling kits and asked to collect saliva on two non-consecutive weekdays. Cortisol measurements were taken at six different time points: immediately after awakening (time point (t)1), 30 min after awakening (t2), 1 h after awakening (t3), at 14:00 (t4), at 16:00 (t5) and immediately before bedtime (t6). Participants documented exact sampling times at each measurement and stored the samples in their freezer until sending them back to the memory clinic. Samples were analyzed at Dresden LabService GmbH (Dresden, Germany). Cortisol levels were determined using a chemiluminescence immunoassay with high sensitivity (IBL International, Hamburg, Germany), with intra-assay and inter-assay CV below 8%. Measurements from the two days were averaged. In the current study, the diurnal cortisol pattern was assessed using two cortisol measures: CAR and cortisol slope. Due to the high number of participants who had invalid t3 data for both measurement days, CAR was calculated without t3. CAR (increase from t1 to t2) was determined as the ‘area under the curve with respect to increase’ [36]. Cortisol slope (wake-to-bed) was calculated as the change between awakening and bedtime cortisol (t6-t1) [37]. Both measures have been previously used in stress research [37].

### CSF sampling

CSF samples were collected by lumbar puncture between the L3/L4 or L4/L5 intervertebral space using a 25-gauge needle. Samples were collected in polypropylene tubes, centrifuged within 2 h, and assessed for Aβ42, t-tau, and p-tau 181 concentrations with a commercially available ELISA (Innogenetics, Ghent, Belgium). CSF Aβ42 levels ≥550 ng/L were considered normal and CSF Aβ42 levels <550 ng/L as pathologic, based on laboratory-recommended cut-off at the Karolinska memory clinic shown to discriminate well between AD and SCD [38]. Additional sample aliquots were stored at –80 °C, until further analyzed.

### CSF biomarkers of neuroinflammation and vascular dysfunction

The ultrasensitive Mesoscale Discovery immunoassay (Mesoscale Diagnostics, Rockville, MD) V-PLEX Human Neuroinflammation Panel 1 was used to assess 37 biomarkers of neuroinflammation, vascular injury, and angiogenesis (https://www.mesoscale.com/en/products/v-plex-neuroinflammation-panel-1-human-kit-k15210d/). These included: c-reactive protein (CRP), eotaxin, eotaxin-3, basic fibroblast growth factor (bFGF), FMS-like tyrosine kinase 1 (Flt-1), vascular cell adhesion molecule-1 (VCAM-1/CD106), intercellular adhesion molecule-1 (ICAM-1/CD54), interferon γ (IFN-γ), IL-1α, IL-1β, IL-2, IL-4, IL-5, IL-6, IL-7, IL-8, IL-10, IL-12/IL-23p40, IL-13, IL-15, IL-16, IL-17A, interferon γ-inducible protein (IP-10), monocyte chemoattractant protein-1 (MCP-1/CCL2), monocyte chemotactic protein-4 (MCP-4/CCL-13), macrophage-derived chemokine (MDC), macrophage inflammatory protein 1α (MIP-1α), macrophage inflammatory protein 1β (MIP-1β), placental growth factor (PlGF), thymus and activation regulated chemokine (TARC/CCL17), tyrosine kinase 2 (Tie-2), serum amyloid A (SAA), TNF-α, TNF-β, vascular endothelial growth factor A (VEGF/VEGF-A), vascular endothelial growth factor C (VEGF-C), vascular endothelial growth factor D (VEGF-D). The procedure was performed according to the manufacturer’s protocol. SCD, MCI, and AD cases were randomly assigned in each plate in approximately equal numbers. Three samples were used as internal controls and were run in all plates. Each array was scanned in an MSD QuickPlex 120 and concentration data was retrieved using Discovery Workbench 4.0. Analytes that were above the lowest limit of detection (LLOD) in 100% of the samples and with <20% intra-assay CV were considered for analysis. This resulted in the inclusion of the following biomarkers: CRP, Flt-1, VCAM-1, ICAM-1, IL-5, IL-6, IL-8, IL-12/IL-23p40, IL-15, IL-16, IP-10, MCP-1, MIP-1β, PlGF, TARC, SAA, VEGF, VEGF-D. Human chitinase 3-like 1 (YKL-40/CHI3L1) was quantified with a commercially available sandwich ELISA kit (DC3L10, R&D Systems, USA), following the manufacturer’s instructions with a sample dilution 1:150. Samples were assayed in duplicate and the average was subsequently used for the statistical analyses. Absorbance was obtained at 450 nm with a microplate reader (Tecan Life Sciences, Männedorf, Switzerland). Protein concentrations were calculated from the standard curves with GraphPad Prism 8.3.1 software using a 4PL curve fit. The calculated intra-assay and inter-assay CV were <10% and <15% respectively.

### CSF synaptic biomarkers

NG was quantified with the prototype NeuroToolKit (Roche Diagnostics International Ltd.) on a cobas e411 or e601 instrument, according to a previously established protocol [8]. SNAP-25 and SYT-1 were determined by mass spectrometry, as published elsewhere [4]. All measurements were performed at the Clinical Neurochemistry Laboratory, Sahlgrenska University Hospital, Mölndal, Sweden.

### Statistical analysis

Descriptive statistics including mean, standard deviation (SD), frequency, and percentages were calculated. Age and education differences between diagnostic groups were assessed by Kruskal-Wallis test due to non-normal distributions. Perceived stress scale (PSS), geriatric depression scale (GDS), and all CSF and salivary biomarkers except IL-8 were non-normally distributed and were therefore transformed with zero skewness logarithmic transformation when tested in analysis of covariance (ANCOVA) and linear regression models. For the correlation heatmap, Spearman’s test was applied using non-transformed (raw) biomarker data. ANCOVA was used for comparisons of continuous biomarker and neuropsychological scales between the groups, covarying for age and sex. Chi square was applied for categorical data.

Principal component analysis (PCA) was used to cluster biomarkers into a few components that could summarize the variance of the data. The following biomarkers were included in the PCA: CRP, Flt-1, VCAM-1, ICAM-1, IL-5, IL-6, IL-8, IL-12/IL-23p40, IL-15, IL-16, IP-10, MCP-1, MIP-1β, PlGF, TARC, SAA, VEGF, VEGF-D, YKL-40/CHI3L1, CAR and cortisol slope. Z scores for each variable were produced and used further in the analysis. A total of 101 participants were included, for whom complete biomarker data were available. The following criteria for the factorability of the correlations were used: 1) a significant Bartlett’s test of sphericity (p < 0.05) 2) Kaiser-Meyer-Olkin (KMO) measure of sampling adequacy for each variable ≥ 0.75, 3) Anti-image correlation matrix diagonals above 0.5, 4) only variables based on factor loadings ≥ |0.5| were considered significant in contributing to the respective factor. The number of extracted components (N = 6) was selected based on eigenvalues > 1 (Supplementary Fig. 1). All the above criteria were fulfilled. Components were non-normally distributed and were transformed with zero skewness logarithmic transformation before being tested in ANCOVAs and linear regression models. Component differences between diagnostic and amyloid pathology groups were analyzed by ANCOVAs, adjusting for age and sex. Separate linear regression models were applied with Aβ42, t-tau, p-tau, SNAP-25, NG, SYT-1, memory, processing speed, PSS, and GDS as outcome measures and each component as a regressor, adjusting for age, sex, and diagnosis. The models for the neuropsychological scales (memory, processing speed, PSS, and GDS) were additionally adjusted for education. In stratified analysis, the same linear regression parameters (excluding diagnosis adjustment) were run in each amyloid pathology status group. The level of significance was set to p < 0.05. Analyses were performed using SPSS Statistics, version 28.0 (IBM Corp, IL, USA). Figures were built using R software. Stata software, version 14 (StataCorp) was used for the zero-skewness logarithmic transformations.

## Results

### Study population characteristics

A total of 108 participants (40 SCD, 39 MCI, and 29 AD) were included in the analysis. Basic demographic, biomarker, and neuropsychological assessments of the study sample are described in Table 1. In brief, AD participants were significantly older (67.7 years old) than SCD (59.6 years old) and MCI (61.5 years old) (p = 0.001) whereas sex distribution and education were similar among the three diagnostic groups. CSF Aβ42 levels were lower (p < 0.001) while t-tau and p-tau levels were higher in AD individuals (p < 0.001 for both), after age adjustment compared to the other two groups. The majority of the AD group was positive for amyloid pathology (86.2%), while 20.5% of the MCI and 2.5% of the SCD groups consisted of amyloid-positive subjects. AD participants performed worse in memory (p < 0.001) and processing speed (p = 0.021) tests compared to SCD and MCI subjects. There were no significant differences between the groups in terms of depressive symptoms and perceived stress assessments.

**Table 1:** Demographic, biomarker, and neuropsychological characteristics of the study population.

	N	SCD (N = 40)	MCI (N = 39)	AD (N = 29)	p
Age	108	59.6 (6.0)	61.5 (7.0)	67.7 (8.3)	**<0.001**
Men/Women, %	108	40.0/60.0	46.2/53.8	40.7/59.3	0.621
Education years	108	14.2 (3.5)	13.6 (3.3)	13.5 (3.0)	0.657
Aβ42, pg/mL	108	861 (163)	727 (193)	430 (106)	**<0.001**
Aβ42, % positive	108	2.5	20.5	86.2	**<0.001**
t-tau, pg/mL	108	262 (111)	309 (1567)	633 (277)	**<0.001**
p-tau, pg/mL	108	39.4 (14.1)	43.4 (17.9)	74.6 (29.8)	**<0.001**
Memory^a^	97	−0.04 (0.84)	−1.3 (1.2)	−2.8 (0.9)	**<0.001**
Processing speed^b^	91	−0.32 (1.1)	−0.94 (1.2)	−1.6 (1.0)	**0.021**
Geriatric Depression Scale (GDS)^c^	101	5.3 (3.7)	6.8 (4.7)	4.9 (3.9)	0.211
Perceived Stress Scale (PSS)^d^	96	26.3 (6.9)	27.2 (10.1)	24.5 (9.5)	0.687

Data are shown as unadjusted mean (SD), unless otherwise stated. P values for age and education were calculated by Kruskal-Wallis test. One-way ANCOVA was applied for analysis of CSF biomarkers and neuropsychological assessments, with age adjustment. Chi square was used for categorical data. P < 0.05 was considered statistically significant and indicated in bold.

^a^Available data for 40 SCD, 33 MCI, 24 AD.

^b^Available data for 39 SCD, 30 MCI, 22 AD.

^c^Available data for 36 SCD, 38 MCI, 27 AD.

^d^Available data for 32 SCD, 37 MCI, 27 AD.

### Biomarkers of stress, synaptic damage, neuroinflammation and vascular dysfunction among diagnostic groups

Salivary cortisol, a measure representing the free circulating levels of the hormone, is routinely used as a biomarker of the HPA axis response to psychosocial stress [39]. In the current study, we measured CAR, which is the increase of cortisol 30 min post-awakening and cortisol slope, reflecting the change in cortisol levels from awakening to bedtime. There were no differences in CAR and cortisol slope between AD, MCI, and SCD patients (Table 2). Next, we compared the levels of several CSF biomarkers of synaptic dysfunction, neuroinflammation and cerebrovascular function across the three diagnostic groups (Table 2). The synaptic markers NG and SNAP-25 were elevated in AD compared to both SCD and MCI participants (p < 0.001 for both markers), in AD versus SCD (p < 0.001 for both), and in AD versus MCI subjects (p < 0.0001 for NG and p < 0.001 for SNAP-25). Of the 38 inflammatory and vascular biomarkers initially analyzed, half of them passed the quality control criteria and were considered for the final analysis. Of these, IP-10, TARC, CRP, ICAM-1 and VCAM-1 exhibited higher concentrations in MCI patients compared to the other two groups (p = 0.030 for IP-10, p = 0.029 for TARC, p = 0.002 for CRP, p = 0.031 for ICAM-1 and p = 0.007 for VCAM-1). In pairwise comparisons, IP-10 was higher in MCI compared to AD (p = 0.019) and SCD (p = 0.040), TARC was higher in MCI versus AD (p = 0.012), CRP was increased in MCI compared to AD (p < 0.001) and SCD (p = 0.012) and ICAM-1 and VCAM-1 were higher in MCI versus AD (p = 0.011 for ICAM-1 and p = 0.002 for VCAM-1). There were no significant differences for the rest of the biomarkers. All comparisons were performed with age and sex adjustments.

**Table 2:** Concentrations of salivary cortisol and CSF synaptic, neuroinflammation and vascular dysfunction markers among diagnostic groups.

	N	SCD (N = 40)	MCI (N = 39)	AD (N = 29)	p
Salivary cortisol					
CAR	106	0.78 (1.5)	0.44 (1.0)	0.11 (1.3)	0.194
Cortisol slope	107	−5.1 (4.1)	−5.1 (11.8)	−9.4 (5.3)	0.105
Synaptic loss					
NG	107	172 (54.1)	176 (76.6)	278 (97.8)^a,b^	**<0.001**
SYT-1	108	33.3 (8.7)	34.5 (10.7)	38.9 (10.4)	0.427
SNAP-25	108	10.8 (1.8)	11.2 (2.1)	14.1 (2.8)^a,c^	**<0.001**
Neuroinflammation					
YKL-40	108	120 (45.0)	145 (65.6)	164 (57.0)	0.427
IL-5	107	0.86 (0.22)	0.87 (0.25)	0.91 (0.27)	0.618
IL-6	106	1.9 (1.3)	1.6 (1.1)	1.4 (0.45)	0.321
IL-8	106	43.5 (9.2)	41.6 (7.7)	43.5 (9.1)	0.388
IL-12/IL-23p40	104	5.6 (2.9)	5.5 (2.3)	5.1 (1.7)	0.551
IL-15	106	3.2 (0.79)	3.4 (1.1)	3.6 (1.1)	0.620
IL-16	106	12.8 (3.4)	13.6 (6.2)	13.3 (3.8)	0.803
MCP-1	106	379 (89.5)	350 (74.5)	394. (89.3)	0.194
MIP-1b	106	15.0 (4.7)	16.1 (6.6)	15.8 (4.7)	0.806
TARC	106	3.9 (1.9)	5.2 (4.4)	3.8 (1.7)^d^	**0.029**
IP-10	106	628 (358)^e^	786 (488)	633 (310)^d^	**0.030**
Vascular dysfunction					
Flt-1	106	14.3 (5.9)	16.3 (6.9)	18.4 (7.6)	0.446
PlGF	106	8.5 (5.5)	9.9 (6.8)	12.3 (8.9)	0.767
VEGF	105	2.7 (0.65)	3.0 (1.1)	3.3 (1.5)	0.806
VEGF-D	106	14.9 (7.7)	15.6 (6.7)	14.9 (6.1)	0.463
CRP	106	3027 (3560)^d^	7974 (12896)	2934 (3812)^c^	**0.002**
ICAM-1	106	1790 (422)	2117 (752)	1943 (562)^d^	**0.031**
SAA	106	904 (1317)	1292 (1453)	893 (838)	0.107
VCAM-1	106	6004 (1391)	7026 (2501)	6250 (1804)^f^	**0.007**

Biomarker levels are shown as unadjusted mean (SD). SYT-1 and SNAP-25 levels are reported in pM, YKL-40 in ng/mL, and the rest biomarkers in pg/mL. P values are calculated from one-way ANCOVA adjusted for age and sex. P < 0.05 was considered statistically significant and indicated in bold. *AD* Alzheimer’s disease, *CAR* cortisol awakening response, *CRP* C-reactive protein, *Flt-1* fms-like tyrosine kinase 1, *ICAM-1* intercellular adhesion molecule-1, *IL-5* interleukin 5, *IP-10* interferon γ-inducible protein, *MCI* mild cognitive impairment, *MCP-1* monocyte chemoattractant protein-1, *MIP-1β* macrophage inflammatory protein *1β*, *NG* neurogranin, *PlGF* placental growth factor, *SAA* serum amyloid A, *SCD* subjective cognitive decline, *SNAP-25* synaptosomal associated protein 25, *SYT-1* synaptotagmin 1, *TARC* thymus and activation regulated chemokine, *VCAM-1* vascular cell adhesion molecule-1, *VEGF* vascular endothelial growth factor, YKL-40, chitinase 3-like 1.

^a^p < 0.001 versus SCD.

^b^p < 0.0001 versus MCI.

^c^p < 0.001 versus MCI.

^d^p < 0.05 versus MCI.

^e^p < 0.05 versus MCI.

^f^p < 0.01 versus MCI.

### Individual biomarker cross-correlations

**Fig. 1:**
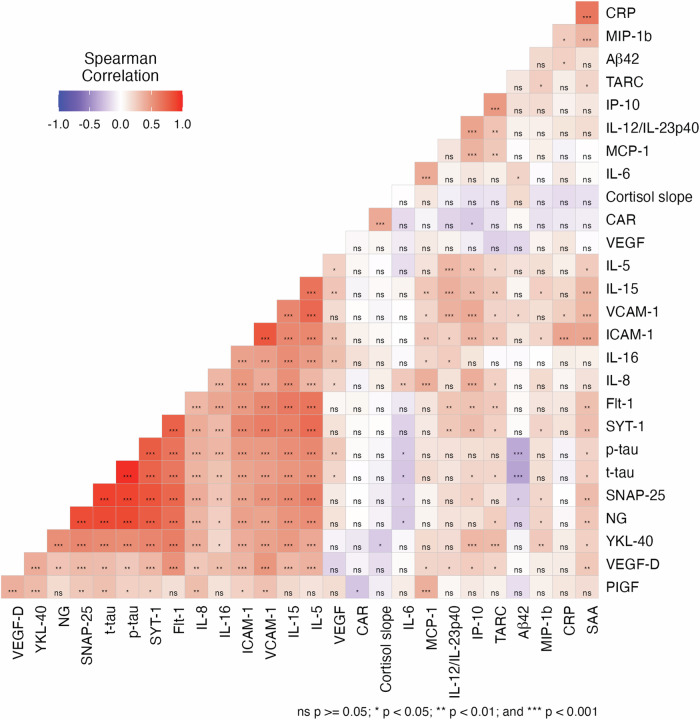
Correlation heatmap of the analysed CSF and diurnal cortisol biomarkers. CSF Aβ42, t-tau, p-tau, and all biomarkers from Table 2 are included in the analysis. Correlation coefficients (Spearman’s test) are indicated in red/blue depending on the direction of the association (red: positive association, blue: negative association). ns p ≥ 0.05, *p < 0.05, **p < 0.01, ***p < 0.001.

Next, we investigated the bivariate correlations of neuroinflammatory, cerebrovascular, and cortisol markers with the traditional AD and synaptic biomarkers in the total population. An overview of the results is presented as a correlation heatmap (Fig. 1). In brief, positive correlations were observed between p-tau, t-tau, synaptic (SNAP-25, NG, SYT-1) and several neuroinflammation (YKL-40, IL-5, IL-8, IL-15, IL-16) and vascular dysfunction markers (Flt-1, ICAM-1, VCAM-1). Aβ42 correlated weakly but significantly with VCAM-1, IL-6, and CRP. CAR correlated negatively with IP-10, PlGF and tended to correlate negatively with IL-12/IL-23p40 (p = 0.07). Finally, an inverse correlation of cortisol slope with YKL-40 was observed.

### Component characteristics and distribution between diagnostic and amyloid pathology status groups

The quantity and variety of the bivariate correlations made the interpretation difficult. Thus, we performed PCA in the total cohort (N = 101) to reduce the number of variables and obtain a few hypothetical components that could maximally explain the variance of the data. Only the more exploratory biomarkers (salivary cortisol, neuroinflammation, and vascular dysfunction) were added in the analyses, as the traditional AD and synaptic markers would have masked the results. This led to the inclusion of 21 biomarkers, with a variable-to-subject ratio of ∼1:5. Applying this method resulted in 6 principal components (PC), explaining cumulatively 68.9% of the variation. The first three components (PC1, PC2, PC3) described 29.1%, 10.2%, and 9.9% of the variation respectively, while the remaining components’ (PC4, PC5, and PC6) contribution was 7.7%, 6.7%, and 6.3% respectively. The factor loading matrix is presented in Supplementary Table 1. PC1 correlated the most with Flt-1, IL-5, VCAM-1, IL-15, YKL-40, ICAM-1, VEGF-D and IL-16, PC2 with IP-10, IL-12/IL-23p40 and TARC, PC3 with CRP, SAA and MIP-1b, PC4 with MCP-1, PlGF, IL-8 and IL-6, PC5 with cortisol slope, IL-6 and CAR and PC6 with VEGF and IL-16.

**Fig. 2:**
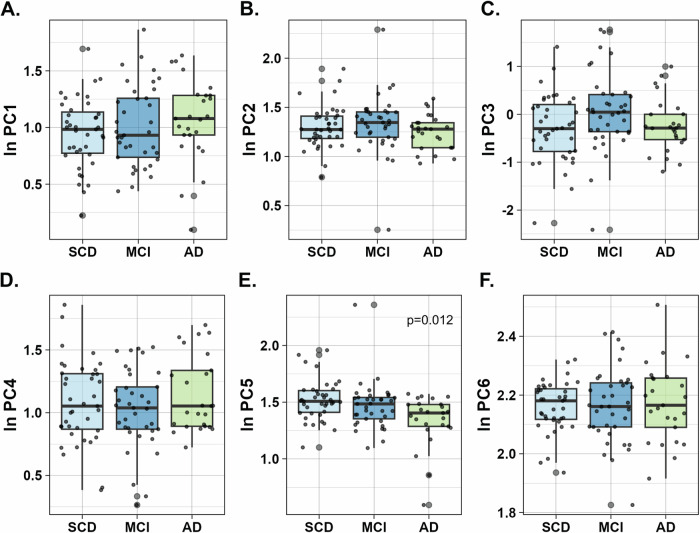
Box-plots depicting component scores among SCD, MCI, and AD patients. **A**–**F** Principal components 1-6 (PC1-PC6). Component scores are in y-axis. The median is shown as a horizontal line, and the whiskers represent the 10th and 90th percentiles. *P* values were calculated by analysis of covariance (ANCOVA), adjusting for age and sex. Only *p*-*values* for significant differences are presented (*P* < 0.05 was considered statistically significant). AD Alzheimer’s disease, MCI mild cognitive impairment, PC1 principal component 1, SCD subjective cognitive decline.

Plotting the coordinates of the observations of the two first components could not visually discriminate either the diagnostic groups (Supplementary Fig. 2A) or the amyloid positive vs negative individuals (Supplementary Fig. 2B). Next, we explored each component’s distribution among diagnostic (Fig. 2) and amyloid status (Supplementary Fig. 3) groups. PC5 was found to be significantly lower in AD compared to SCD and MCI participants (p = 0.012) (Fig. 2E) and tended to be decreased in the amyloid-positive group (p = 0.089) (Supplementary Fig. 3E). PC2 was also decreased in subjects positive for amyloid pathology (p = 0.011) (Supplementary Fig. 3B). No other differences could be observed for the rest of the components.

### Associations of the principal components with CSF biomarkers of Alzheimer’s disease pathology and synaptic damage

**Fig. 3:**
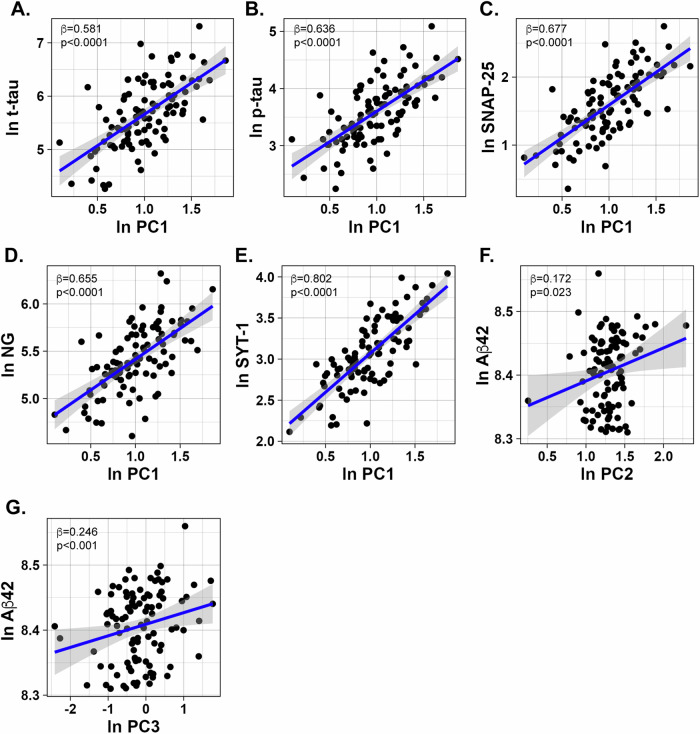
Associations of components with CSF biomarkers of AD pathology and synaptic damage. Scatter plots representing associations of principal component 1 (PC1) with **A** t-tau, **B** p-tau, **C** SNAP-25, **D** NG, **E** SYT-1 and associations of **F** principal component 2 (PC2) with Aß42 and **G** principal component 3 (PC3) with Aß42. Scatter plots representing associations of PC1 with t-tau, p-tau, SNAP-25, NG, and SYT-1 and associations of PC2 and PC3 with Aβ42. The standardized regression coefficients (*β*) and *p* values shown were computed using linear regression models adjusting for age, sex, and diagnosis.

We next investigated possible associations of each component with CSF markers of AD pathology and synaptic damage in the total cohort, by linear regression models (Supplementary Table 2). All models were adjusted for age, sex and diagnosis. PC1 associated with t-tau (β = 0.58, p < 0.0001), p-tau (β = 0.64, p < 0.0001) and all markers of synaptic dysfunction (β = 0.68, p < 0.0001 for SNAP-25, β = 0.66, p < 0.0001 for NG and β = 0.80, p < 0.0001 for SYT-1) (Supplementary Table 2 and Fig. 3A–E). In addition, PC2 and PC3 associated positively with Aβ42 levels (β = 0.17, p = 0.023 for PC2 and β = 0.22, p < 0.001 for PC3) (Supplementary Table 2 and Fig. 3F, G). A trend was observed for a positive association of PC1 with Aβ42 levels (β = 0.16, p = 0.056).

Next, we conducted the same analysis after stratifying participants based on their amyloid status. The associations of PC1 with tau and synaptic markers remained significant irrespective of amyloid status (Supplementary Table 3). Interestingly, PC1 and PC3 were associated with increased Aβ42 levels in the group negative to amyloid β pathology (β = 0.35, p = 0.014 for PC1 and β = 0.25, p = 0.044 for PC3) but not in amyloid positive participants. Finally, no association could be observed for PC2 and Aβ42 levels in any of the two groups of amyloid β pathology.

### Associations of principal components with cognition, depressive symptoms, and perceived stress

In a next step, we sought to investigate associations between components and memory, processing speed, depressive symptoms, and perceived stress. To this end, we performed separate linear regression analyses in the total population with each assessment as the outcome measure and each of the principal components as the regressor, adjusting for age, sex, education, and diagnosis. PC4 was associated with a worsened processing speed (β = −0.21, p = 0.047). No other associations were observed for the rest of the components (Table 3).

**Table 3:** Associations of principal components with neuropsychological assessments.

	Memory β (p)	Processing speed β (p)	PSS β (p)	GDS β (p)
PC1	0.11 (0.164)	0.07 (0.521)	0.03 (0.791)	0.03 (0.793)
PC2	−0.12 (0.108)	0.00(0.989)	−0.04 (0.706)	−0.02 (0.844)
PC3	0.00 (0.958)	−0.07 (0.492)	0.07 (0.531)	0.12 (0.227)
PC4	−0.11 (0.164)	−**0.21 (0.047)**	0.07 (0.545)	0.03 (0.752)
PC5	0.10 (0.157)	−0.14 (0.156)	0.01 (0.957)	0.01 (0.934)
PC6	0.01 (0.883)	0.03 (0.778)	0.02 (0.835)	0.06 (0.527)

Results are from separate linear regression models with memory, processing speed, perceived stress (PSS), or depressive symptoms (GDS) as outcome measures and each component as a regressor. Data are shown as standardized β coefficients (p values), after age, sex, education, and diagnosis adjustment. P < 0.05 was considered statistically significant and indicated in bold. *GDS* geriatric depression scale, *PC1* principal component 1, *PSS* perceived stress scale.

When stratifying for amyloid β pathology status, a trend for PC4 and lower processing speed was observed in the amyloid positive group only (β = −0.40, p = 0.064) (Supplementary Table 4). Further, PC2 was related to worsened memory only in amyloid-positive participants (β = −0.47, p = 0.012). An association of PC3 with depressive symptoms was also observed in the amyloid-positive group (β = 0.45, p = 0.022). No other relationships could be seen for the rest of the analyses.

## Discussion

Previous studies on HPA axis dysfunction or neuroinflammation in AD, have focused mainly on single biomarkers or a small set of factors. By using a multi-biomarker approach, we explored here the relationship of AD with diurnal cortisol and a broad panel of neuroinflammatory and cerebrovascular biomarkers, in a well-characterized cohort from a real-life memory clinic setting. We found increased CSF levels of CRP, IP-10, TARC, ICAM-1, and VCAM-1 in MCI patients. Further, markers of cortisol dysregulation (flattened saliva CAR and cortisol slope) correlated with increased levels of PlGF, IP-10, and YKL-40. A biosignature composed of diurnal cortisol and IL-6 was downregulated in AD patients. Finally, neuroinflammatory and cerebrovascular injury biosignatures were associated with AD pathology, synaptic failure, and worsened processing speed.

Several studies have investigated the role of neuroinflammatory and cerebrovascular dysfunction factors over the course of AD, yet often presenting inconclusive or contradictory results [40]. However, a trend is found towards an upregulation of cytokines during early AD stages [22, 40]. Herein, we demonstrate that five markers (IP-10, TARC, CRP, ICAM-1, and VCAM-1) are increased at the MCI stage in support of an early microglia and astrocyte activation scenario. All except CRP, correlated with the glia activation markers YKL-40 and MCP-1, further underpinning this scenario. In addition, existing experimental evidence has demonstrated a relationship or even a causative role in microglia activation and cerebrovascular dysfunction for these five proteins [41–46]. Interestingly, recent evidence suggests that microglia activation is associated with higher gray matter and hippocampal volume in MCI subjects [24] and predicts better clinical and cognitive outcomes in AD patients [47–49]. Thus, these findings strengthen the concept of a homeostatic neuroinflammatory response at the early disease states [20, 22]. Our results are consistent with Schuitemaker et al. [50] who reported elevated CSF and serum CRP levels in MCI patients compared to AD individuals. In addition, Brosseron et al. [51] showed that CSF CRP levels decreased from non-demented to MCI and AD participants and correlated with less amyloid pathology. Several studies have investigated the levels of IP-10 in the CSF of AD patients and have found mixed results. Some have reported that IP-10 levels are increased in AD patients compared to controls, while others have found no significant difference between the two groups [40]. Notably, Galimberti et al. demonstrated higher CSF IP-10 levels in patients with MCI and mild AD but not in patients with severe AD [52]. ICAM-1 and VCAM-1 are cell adhesion molecules reflecting vascular damage and blood-brain barrier dysfunction [53]. Previous investigations have shown also conflicting results on their link with AD; Nielsen et al., reported lower CSF levels in AD subjects [54], while Janelidze et al. [30], demonstrated upregulation in preclinical and prodromal AD. Compared to our cohort, both studies had larger populations and older participants. The elevated levels of these markers seen in MCI patients in the present study could be the result of increased BBB permeability. Determining the CSF/serum albumin ratio would allow us to address this hypothesis, but unfortunately, this information was unavailable. Hence, further work will be needed including larger cohorts and longitudinal data to obtain a comprehensive picture of neuroinflammation and vascular dysfunction in the disease pathophysiology.

One aim of this study was to identify combined biomarker signatures including stress, inflammation, and vascular injury, that could separate cognitive disorder phenotypes. The large number of biomarkers investigated prompted us to use a data-driven approach, i.e. principal components analysis. This resulted in a six-component solution explaining 69% of the data variance. Using this model, we did not observe a clear separation of our participants by clinical diagnosis or amyloid β pathology status. This could be partially attributed to the large heterogeneity observed in AD in terms of clinical features, neuropathological profiles, and other confounding factors [55, 56]. When exploring each component individually, we found that component 5 was lower in AD participants and tended to be decreased in amyloid-positive participants. The key contributors of this component were salivary CAR, cortisol slope, and CSF IL-6. A flattened CAR and cortisol slope have been associated to chronic stress and seem to predict cognitive decline and hippocampal atrophy in middle-aged and older healthy adults [15, 57]. However, in AD populations, there are very limited data on diurnal cortisol markers and even fewer on the inter-relationship of cortisol with neuroinflammatory/vascular markers. Herein, we found a correlation of a flattened salivary CAR with the vascular injury factor PlGF and the chemokine IP-10. In addition, a flattened cortisol slope correlated with increased levels of the glia activation marker YKL-40. Thus, these findings support the tight connection of specific aspects of stress response and inflammation within the central nervous system [25].

Of note, IL-6 had a different pattern from most other neuroinflammatory markers in our cohort; increased IL-6 correlated with less AD pathology, neurodegeneration, and synaptic loss, however, there were no differences between the clinical groups. Previous human studies have led to inconsistent results with most reports showing increased or unchanged levels in AD patients [40]. There is also inconclusive evidence regarding IL-6 correlation with AD pathology biomarkers [58–62]. Methodological differences and population characteristics, such as disease stage, the presence of immunological disorders, and genetic and lifestyle factors could account for these discrepancies. The mechanistic link between the relation seen in our study is currently unknown, although it can be hypothesized that it could reflect IL-6 actions that differ from the well-known pro-inflammatory functions of this cytokine. IL-6 is a pleiotropic cytokine involved in other biological processes in the central nervous system, including neuronal development and differentiation, neurogenesis, and amyloid plaque clearance [63–65].

A major finding of our study was the strong correlation of several markers of neuroinflammation (YKL-40, IL-5, IL-8, IL-15, IL-16) and cerebrovascular dysfunction (Flt-1, ICAM-1, VCAM-1) with CSF biomarkers of tau pathology, neurodegeneration, and synaptic loss in the total cohort. These results were further verified by principal component analysis, where we found that component 1, a biomarker signature of Flt-1, IL-5, VCAM-1, IL-15, YKL-40, ICAM-1, VEGF-D and IL-16 showed significant associations with t-tau, p-tau and synaptic markers (SNAP-25, SYT-1, NG). Our findings agree with previous investigations demonstrating correlations of several immune response markers with tau pathology along the AD continuum as well as in cognitively intact older adults [49, 51]. In fact, it has been reported in both animal and human studies that neuroinflammation drives tau pathology in synergy with amyloid β [66–68]. A limited number of studies have explored the association of neuroinflammatory factors with markers of synaptic loss, showing mainly positive correlations in preclinical AD patients [69] and in healthy older adults at risk for dementia [70]. These findings are supported by previous work in animal studies, illustrating that immune-related mechanisms mediate synaptic loss at early AD stages through phagocytosis of synapses [71, 72].

Interestingly, component 2 was decreased in amyloid-positive subjects, while both component 2 and component 3 were related to less amyloid pathology. The main contributors of component 2 were the neuroinflammation markers IP-10, IL-12/IL23p40, and TARC, while component 3 drivers were the cerebrovascular dysfunction biomarkers CRP, SAA, and the chemokine MIP-1β. We can assume that IP-10, TARC, and CRP may be driving these findings as they were elevated in MCI, a group largely composed of amyloid-negative patients in our sample ( ~ 80%).

We identified an association of component 4 with worsened processing speed in the total cohort. The main contributors of component 4 were the chemokine MCP-1, produced by microglia and astrocytes, PlGF, and the pro-inflammatory cytokine IL-8. To date, there are no studies on the relationship of these markers with processing speed in the context of AD. CSF MCP-1 has been reported to be positively associated with MMSE score in AD patients [52] whereas it was shown to predict a faster cognitive decline in prodromal AD subjects [73]. A recent study did not find associations of MCP-1, PlGF, and IL-8 with cognitive performance in a large cohort of cognitively unimpaired MCI participants [74]. We did not observe any relationship of the biomarker components with memory, depressive symptoms, and perceived stress in the total sample. In stratified analysis, we found an inverse relationship of component 2 with memory and a positive association of component 3 with depression in amyloid-positive individuals. However, these findings should be interpreted with caution due to the limited sample size of this group. Taken together, the relationship of neuroinflammatory factors with cognitive performance and depression found here should be further validated in future studies.

Limitations of this work include the relatively small sample size and its exploratory design, which did not allow us to correct for multiple comparisons. Thus, the results should be interpreted with caution. This was a cross-sectional study and therefore the directionality of the relationships found here cannot be addressed. Participants were recruited from a Swedish memory clinic and the findings may not be generalizable to other populations.

In conclusion, we studied the relationship between salivary cortisol and biomarkers of neuroinflammation and cerebrovascular dysfunction in memory clinic patients. Several biomarkers (i.e. CRP, IP-10, TARC, ICAM-1, and VCAM-1) were found to be elevated in the CSF of MCI patients but not in AD, suggesting microglia activation at an early predementia stage. Indicators of cortisol dysregulation (flattened CAR and cortisol slope) correlated with specific neuroinflammation and vascular injury factors. Using a data-driven approach, we found biomarker signatures reflecting overlapping pathophysiological processes of neuroinflammation/vascular injury and HPA-axis dysfunction to be altered in AD patients. We further demonstrated associations of several biosignatures with key features of AD pathology, synaptic failure, and cognitive performance. With regard to the large inter-individual heterogeneity of memory clinic patients, our data suggest that the use of multiple biosignatures could be superior to single biomarkers in capturing changes in these biological processes in AD. Further studies with larger sample sizes and longitudinal data are warranted to evaluate our findings’ potential in tracking patients who could benefit the most from interventions targeting these pathways.

## Supplementary information

Supplementary information

## Data Availability

Anonymized data will be shared with qualified investigators who have Institutional Review Board approval and a Material Transfer Agreement on request.
