# Supplementary material for: Neuroinflammation, cerebrovascular dysfunction and diurnal cortisol biomarkers in a memory clinic cohort: Findings from the Co-STAR study
Source: Transl Psychiatry. 2024 Sep 9;14:364. doi: 10.1038/s41398-024-03072-x (PMC11385239; doi:10.1038/s41398-024-03072-x)
Supplement: Supplementary file 1 — Supplementary information [file 41398_2024_3072_MOESM1_ESM.pdf]

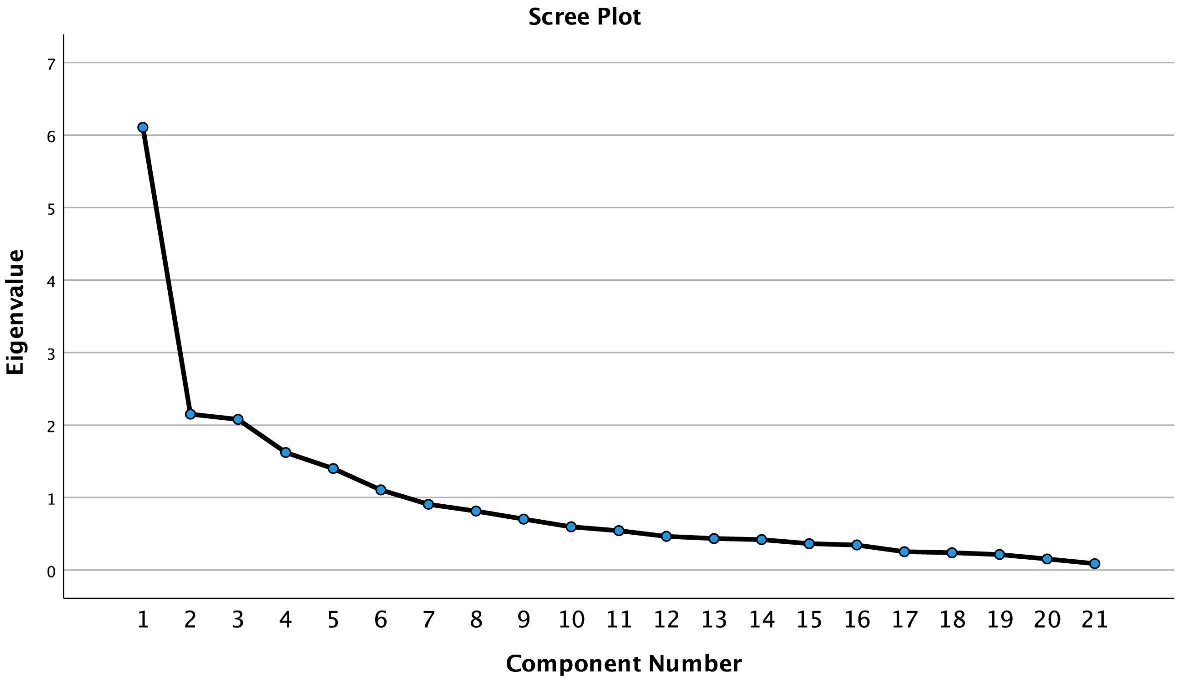

Supplementary Fig. 1. Scree plot of principal component analysis. Scree plot showing the variance explained by each component. The optimum number of components was selected based on Eigen values >1.

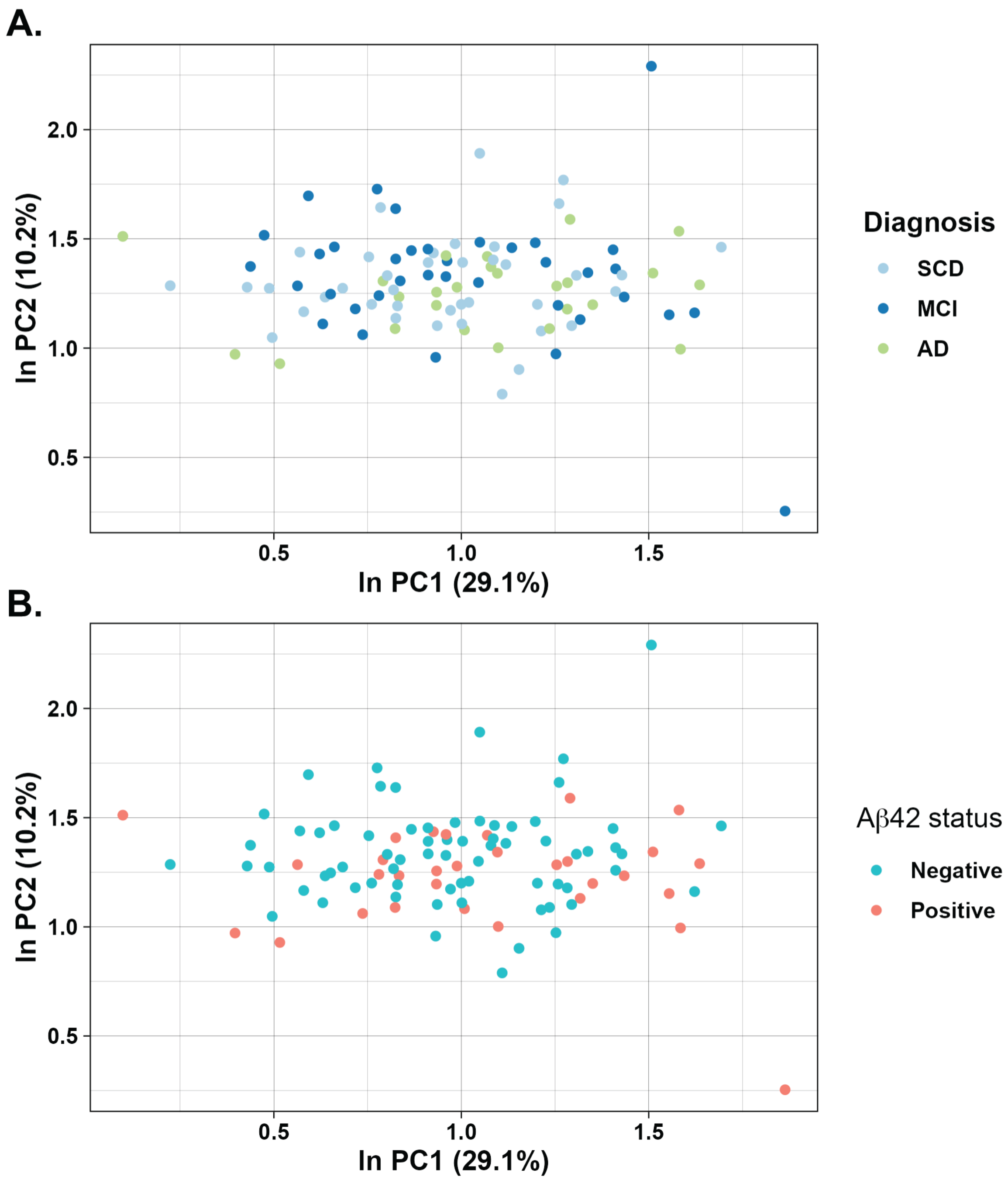

Supplementary Fig. 2. Principal component analysis of diurnal cortisol and CSF inflammatory proteins in diagnostic (A) and amyloid pathology (B) groups. Plots showing coordinates of the observations resulting from the two-component solution, accounting for 29.1% and 10.2% of the variance in the dataset, respectively. Abbreviations: AD, Alzheimer's disease; MCI, mild cognitive impairment; PC1, principal component 1; SCD, subjective cognitive decline.

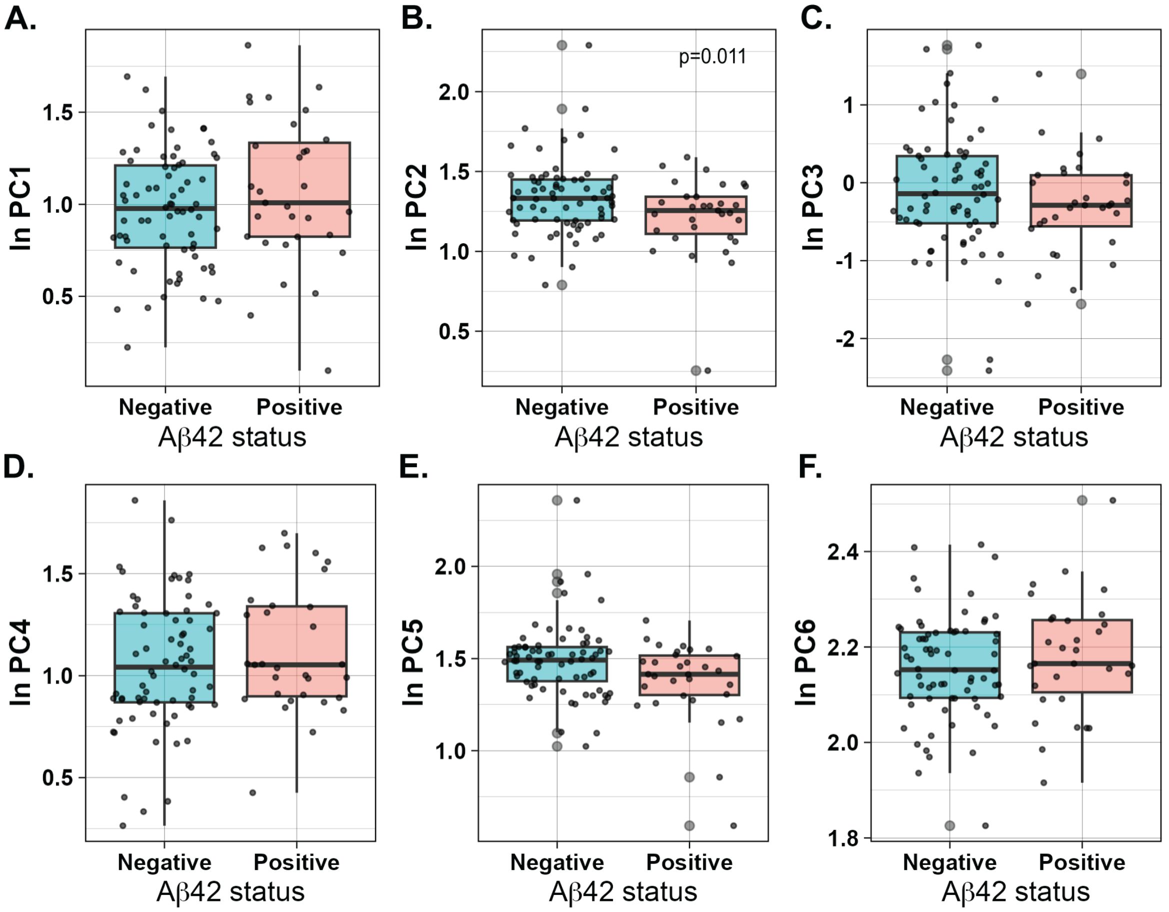

Supplementary Fig. 3. Box-plots depicting component scores among amyloid pathology groups. Component scores are in y-axis. Median shown as horizontal line, whiskers represent 10th and 90th percentiles. P values were calculated by ANCOVA adjusting for age and sex. Only p values for significant differences are presented ($P<0.05$ was considered statistically significant). Abbreviations: amyloid β 42; PC1, principal component 1.

Supplementary Table 1. Factor loading matrix based on a principal component analysis of 21 biomarkers in 101 samples.

	PC1	PC2	PC3	PC4	PC5	PC6
**Flt-1**	.851					
**IL-5**	.828					
**VCAM-1**	.812					
**IL-15**	.792					
**YKL-40**	.738					
**ICAM-1**	.722					
**VEGF-D**	.613					
**IL-16**	.557					.503
**IP-10**		.834				
**IL-12/23p40**		.760				
**TARC**		.759				
**CRP**			.883			
**SAA**			.757			
**MIP-1b**			.617			
**MCP-1**				.798		
**PlGF**				.648		
**IL-8**				.534		
**Cort slope**					.806	
**IL-6**				.503	.671	
**CAR**					.550	
**VEGF**						.805

Only biomarkers based on factor loadings ≥ |0.5| were considered significant in contributing to the respective factor. Abbreviations: CAR, cortisol awakening response; CRP c-reactive protein, Flt-1, fms-like tyrosine kinase 1; ICAM-1, intercellular adhesion molecule-1; IL-5, interleukin 5; IP-10, interferon γ-inducible protein; MCP-1, monocyte chemoattractant protein-; MIP-1β, macrophage inflammatory protein 1β; PC1, principal component 1; PlGF, placental growth factor; SAA, serum amyloid A; TARC, thymus and activation regulated chemokine;

VCAM-1, vascular cell adhesion molecule-1; VEGF, vascular endothelial growth factor; YKL-40, Chitinase 3-like 1.

Supplementary Table 2. Associations of principal components with CSF biomarkers of Alzheimer's disease pathology and synaptic damage.

	**Aβ42 β (p)**	**t-tau β (p)**	**p-tau β (p)**	**SNAP-25 β (p)**	**NG β (p)**	**SYT-1 β (p)**
**PC1**	0.16 (0.056)	**0.58 (<0.0001)**	**0.64 (<0.0001)**	**0.68 (<0.0001)**	**0.66 (<0.0001)**	**0.80 (<0.0001)**
**PC2**	**0.17 (0.023)**	-0.01 (0.866)	-0.02 (0.795)	-0.01 (0.918)	-0.02 (0.816)	0.05 (0.592)
**PC3**	**0.22 (<0.001)**	-0.03 (0.740)	-0.03 (0.692)	0.01 (0.917)	0.13 (0.146)	0.05 (0.631)
**PC4**	-0.01 (0.900)	0.01 (0.946)	-0.06 (0.482)	-0.04 (0.691)	-0.10 (0.306)	-0.08 (0.431)
**PC5**	0.07 (0.347)	0.02 (0.805)	0.06 (0.523)	0.02 (0.861)	0.05 (0.616)	0.06 (0.593)
**PC6**	-0.01 (0.864)	-0.03 (0.740)	0.07 (0.382)	-0.08 (0.340)	0.05 (0.503)	-0.02 (0.844)

Results are from separate linear regression models with Aβ42, t-tau, p-tau, SNAP-25, NG or SYT-1 as outcome measure and each component as regressor. Data are shown as standardized β coefficients (p values), after age, sex and diagnosis adjustment. $P<0.05$ was considered statistically significant. Abbreviations: Aβ42, amyloid β 42; NG, neurogranin; PC1, principal component 1; p-tau, phospho-tau; SNAP-25, synaptosomal associated protein 25; SYT-1, synaptotagmin 1; t-tau, total tau.

Supplementary Table 3. Associations of principal components with CSF biomarkers of Alzheimer's pathology and synaptic damage stratified by amyloid β pathology.

	**Aβ42**		**t-tau**		**p-tau**	
	**Aβ42 negative β (p)**	**Aβ42 positive β (p)**	**Aβ42 negative β (p)**	**Aβ42 positive β (p)**	**Aβ42 negative β (p)**	**Aβ42 positive β (p)**
**PC1**	**0.35 (0.014)**	0.34 (0.087)	**0.66 (<0.0001)**	**0.67 (<0.001)**	**0.69 (<0.0001)**	**0.72 (<0.0001)**
**PC2**	0.18 (0.157)	-0.24 (0.222)	0.20 (0.075)	-0.23 (0.259)	0.18 0.121	-0.23 (0.264)
**PC3**	**0.25 (0.044)**	0.21 (0.268)	-0.04 (0.750)	0.11 (0.590)	-0.06 (0.599)	0.11 (0.573)
**PC4**	0.04 (0.786)	-0.10 (0.642)	-0.08 (0.501)	0.25 (0.259)	-0.14 (0.235)	0.11 (0.608)
**PC5**	0.22 (0.091)	0.34 (0.066)	0.10 (0.399)	-0.20 (0.301)	0.10 (0.410)	-0.06 (0.747)
**PC6**	0.02 (0.848)	0.27 (0.172)	0.01 (0.904)	-0.16 (0.424)	0.12 (0.283)	-0.01 (0.955)
	**SNAP-25**		**NG**		**SYT-1**	
**PC1**	**0.66 (<0.0001)**	**0.74 (<0.0001)**	**0.54 (0.000006)**	**0.81 (<0.0001)**	**0.75 (<0.0001)**	**0.83 (<0.0001)**
**PC2**	0.11 (0.323)	-0.13 (0.529)	0.15 (0.204)	-0.26 (0.214)	0.23 (0.052)	-0.32 (0.101)
**PC3**	-0.02 (0.867)	0.10 (0.616)	0.14 (0.211)	0.10 (0.616)	0.01 (0.952)	0.16 (0.400)
**PC4**	-0.10 (0.384)	0.13 (0.546)	-0.11 (0.373)	-0.03 (0.899)	0.15 (0.475)	-0.06 (0.787)
**PC5**	0.06 (0.626)	-0.13 (0.483)	0.10 (0.396)	-0.10 (0.598)	0.06 (0.622)	-0.01 (0.975)
**PC6**	-0.08 (0.448)	-0.10 (0.608)	-0.01 (0.923)	-0.18 (0.390)	-0.03 (0.797)	-0.03 (0.897)

Results are from separate linear regression models with Aβ42, t-tau, p-tau, SNAP-25, NG or SYT-1 as outcome measure and each component as regressor, stratified by amyloid pathology status. Data are shown as standardized β coefficients (p values), after age and sex adjustment.

P<0.05 was considered statistically significant. Abbreviations: Aβ42, amyloid β 42; NG, neurogranin; PC1, principal component 1; p-tau, phospho-tau; SNAP-25, synaptosomal associated protein 25; SYT-1, synaptotagmin 1; t-tau, total tau.

Supplementary Table 4. Associations of principal components with neuropsychological assessments stratified by amyloid β pathology.

	Memory		Processing speed		GDS		PSS	
	**Aβ42 negative β (p)**	**Aβ42 positive β (p)**	**Aβ42 negative β (p)**	**Aβ42 positive β (p)**	**Aβ42 negative β (p)**	**Aβ42 positive β (p)**	**Aβ42 negative β (p)**	**Aβ42 positive β (p)**
**PC1**	0.06 (0.691)	0.35 (0.079)	0.15 (0.280)	-0.09 (0.617)	0.04 (0.752)	-0.12 (0.566)	0.05 (0.701)	-0.02 (0.926)
**PC2**	-0.13 (0.297)	**-0.47 (0.012)**	-0.04 (0.771)	-0.05 (0.789)	0.11 (0.319)	-0.27 (0.193)	0.01 (0.919)	-0.18 (0.406)
**PC3**	-0.15 (0.186)	0.06 (0.771)	-0.19 (0.113)	0.21 (0.268)	0.06 (0.581)	**0.45 (0.022)**	0.03 (0.811)	0.29 (0.160)
**PC4**	-0.02 (0.852)	-0.20 (0.328)	-0.16 (0.188)	-0.40 (0.064)	-0.12 (0.315)	0.14 (0.525)	-0.05 (0.665)	0.12 (0.578)
**PC5**	0.23 (0.057)	0.24 (0.209)	-0.18 (0.146)	0.19 (0.294)	-0.05 (0.660)	-0.04 (0.825)	-0.01 (0.911)	-0.06 (0.766)
**PC6**	-0.06 (0.602)	0.16 (0.396)	-0.04 (0.723)	0.16 (0.404)	-0.05 (0.639)	0.23 (0.238)	-0.19 (0.100)	0.36 (0.080)

Results are from separate linear regression models with memory, processing speed, GDS or PSS as outcome measure and each component as regressor. Data are shown as standardized β coefficients (p values), after age, sex and education adjustment. P<0.05 was considered statistically significant. Abbreviations: GDS, geriatric depression scale; PC1, principal component 1; PSS, perceived stress scale.
